# Acral Lentiginous Melanoma: A Rare Variant With Unique Diagnostic Challenges

**DOI:** 10.7759/cureus.8424

**Published:** 2020-06-03

**Authors:** Brett C Brazen, Taylor Gray, Maheera Farsi, Richard Miller

**Affiliations:** 1 Dermatology, Nova Southeastern University - Kiran C. Patel College of Osteopathic Medicine, Palm Harbor, USA; 2 Dermatology, Largo Medical Center, Largo, USA; 3 Dermatology, Hospital Corporation of America/University of South Florida, Morsani College of Medicine, Largo Medical Center Program, Largo, USA; 4 Dermatology, Bay Dermatology and Cosmetic Surgery, Largo Medical Center, Largo, USA

**Keywords:** malignant melanoma, cutaneous oncology, dermatology, dermatopathology, genodermatoses

## Abstract

Acral lentiginous melanoma (ALM), named for its location and histological growth pattern, is a rare variant of melanoma. ALM presents on palms, soles, or in association with the nail unit. While ALM accounts for approximately 5% of melanomas diagnosed each year, it is the most commonly diagnosed subtype of melanoma in non-Caucasian patients, and it is most likely to be diagnosed in the seventh decade of life. We present a case of a 72-year-old, Fitzpatrick skin type (FST) 5 female who presented to our clinic with a chief complaint of a slowly enlarging dark brown patch with a variation of pigment changes that had been present for 10 years on her right plantar surface. Biopsy obtained for hematoxylin and eosin (H&E) revealed malignant melanoma in situ, acral lentiginous type. Here, we will discuss the unique pathogenesis of ALM, as well as, its characteristic clinical and histological findings. Furthermore, this case underscores the importance of physician and patient education to raise awareness of this rare type of melanoma, specifically in patients with skin of color in hopes of decreasing time to diagnosis and improving prognosis.

## Introduction

Acral lentiginous melanoma (ALM) is a relatively uncommon type of cutaneous melanoma that occurs on palms, soles, or in association with the nail apparatus. ALM is most commonly diagnosed in the seventh decade of life and accounts for approximately 5% of all melanomas [1]. As darkly pigmented individuals are less likely to develop melanomas related to ultraviolet (UV) exposure, ALM represents a disproportionate percentage of melanomas diagnosed in darker-skinned patients [1]. However, the incidence of ALM is similar across racial and ethnic groups [1]. This report is intended to educate the medical community about the rarity of this condition and to help physicians of all specialties accurately diagnose and treat ALM.

## Case presentation

**Figure 1:**
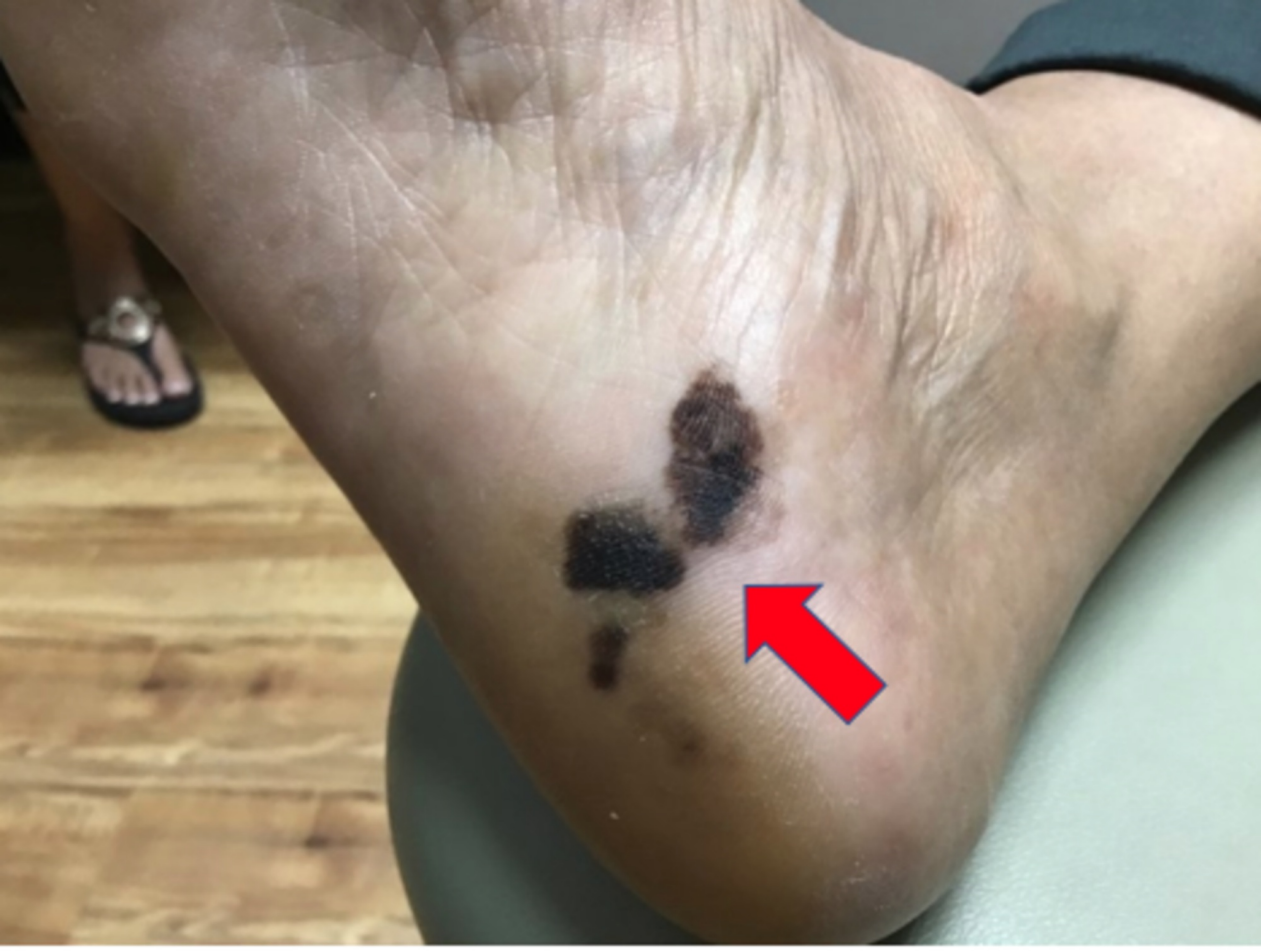
A 3.0 cm x 1.5 cm well-demarcated, brown to black patch with two adjacent smaller brown to black patches with scalloped borders on the right plantar surface.

We present a 72-year-old, Fitzpatrick skin type (FST) 5 female, with a past medical history of insulin-dependent diabetes mellitus II and hypertension who came to our dermatology clinic with a concern of an enlarging dark spot on the plantar surface of her right foot. The patient stated she first noticed the lesion 10 years ago, but it had been rapidly enlarging and darkening for the past three years. The patient has no personal or family history of melanoma. Furthermore, in the past three years, two smaller brown to black patches became apparent adjacent to the original lesion. The patient denied pain, pruritus, bleeding, or any other symptoms associated with the lesion or any systemic symptoms including fevers, chills, unintentional weight loss, cough, and headache. On physical exam, a 3.0 cm x 1.5 cm well-demarcated, brown to black patch with two adjacent smaller brown to black patches, all with scalloped borders, were noted on the right plantar surface (Figure 1).

**Figure 2:**
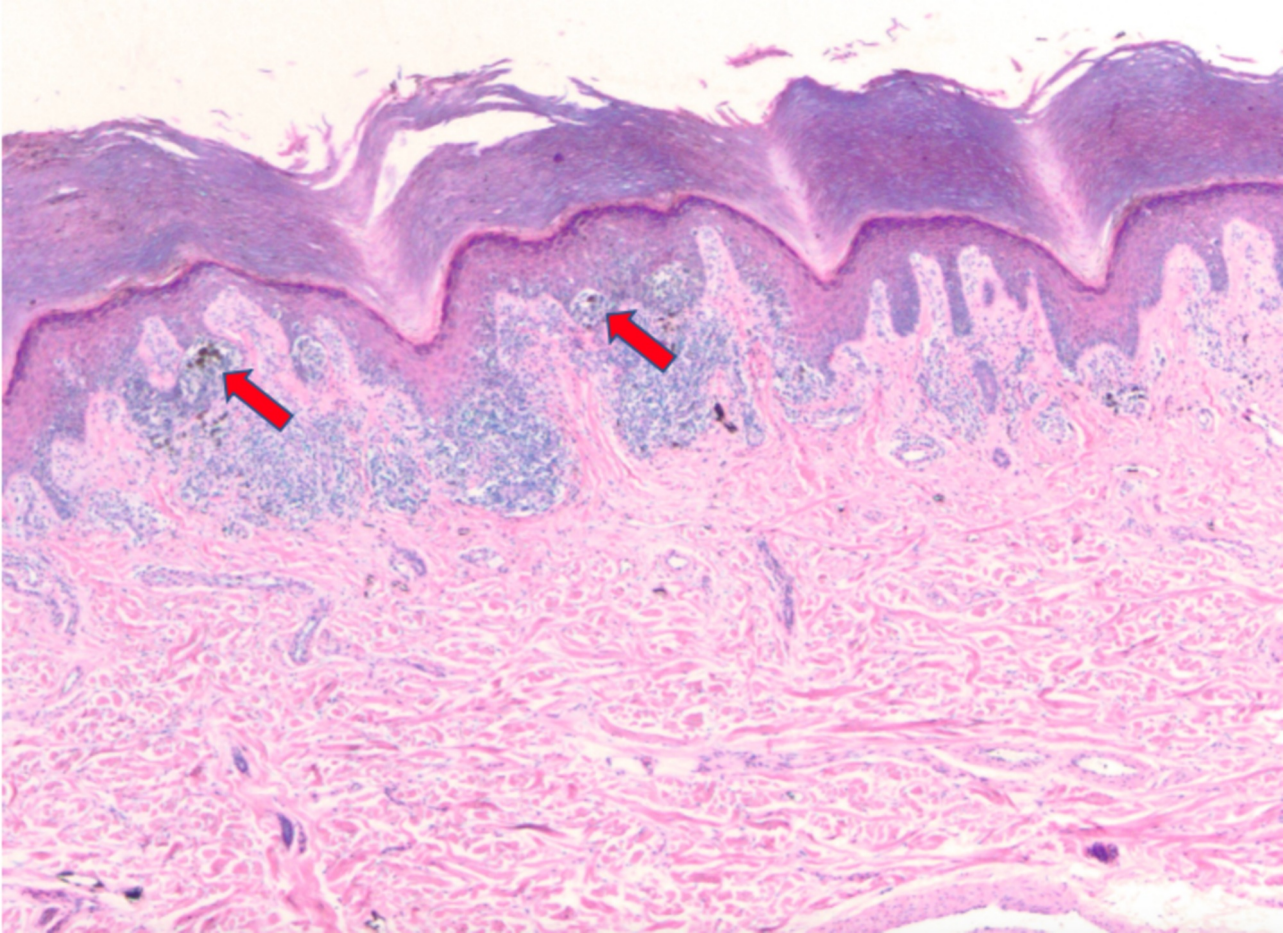
Extensive proliferation of malignant melanocytes in a nested array (4x).

**Figure 3:**
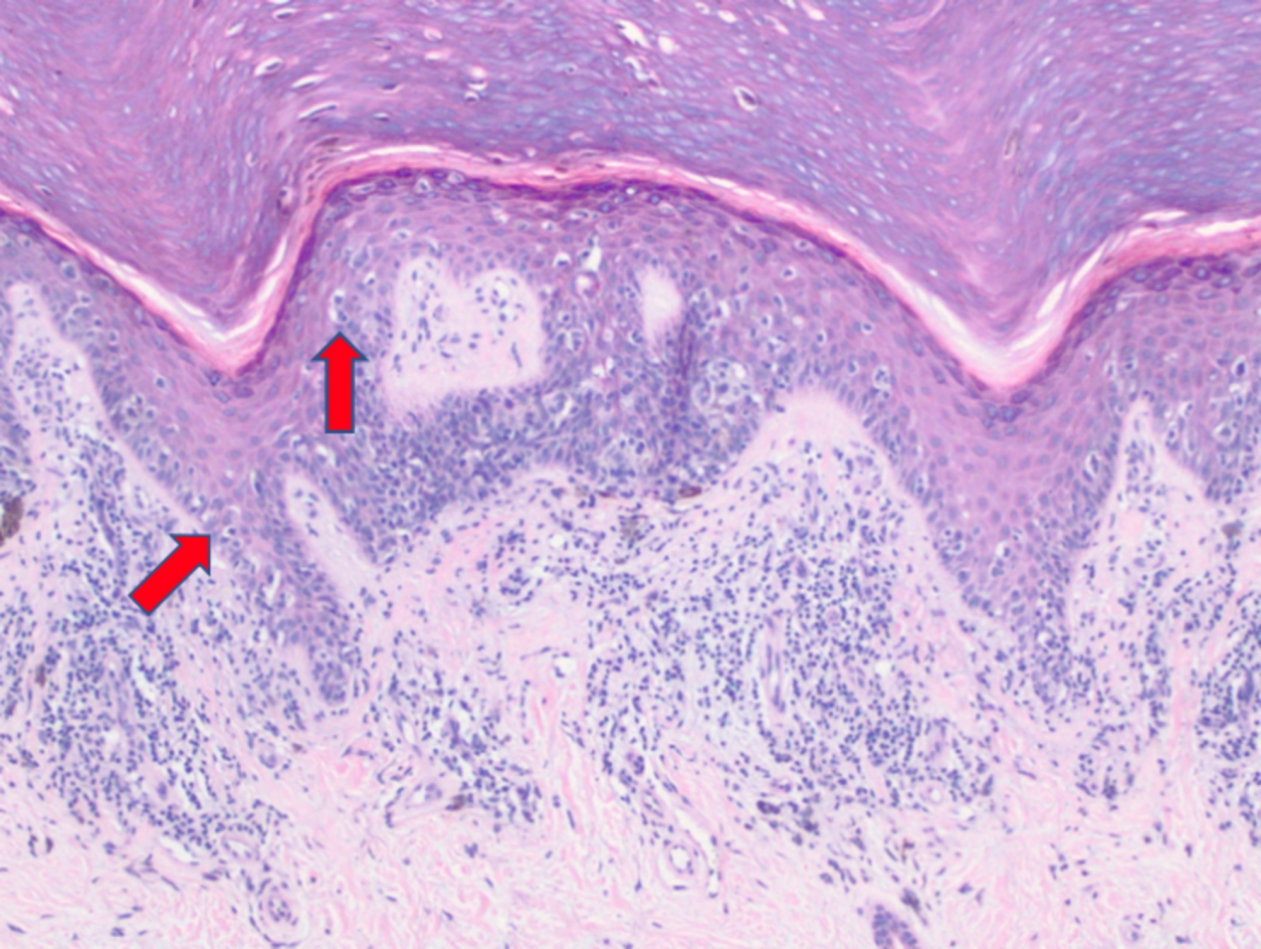
Extensive proliferation of malignant melanocytes in a confluent (left arrow), and pagetoid array (right arrow) (10x).

**Figure 4:**
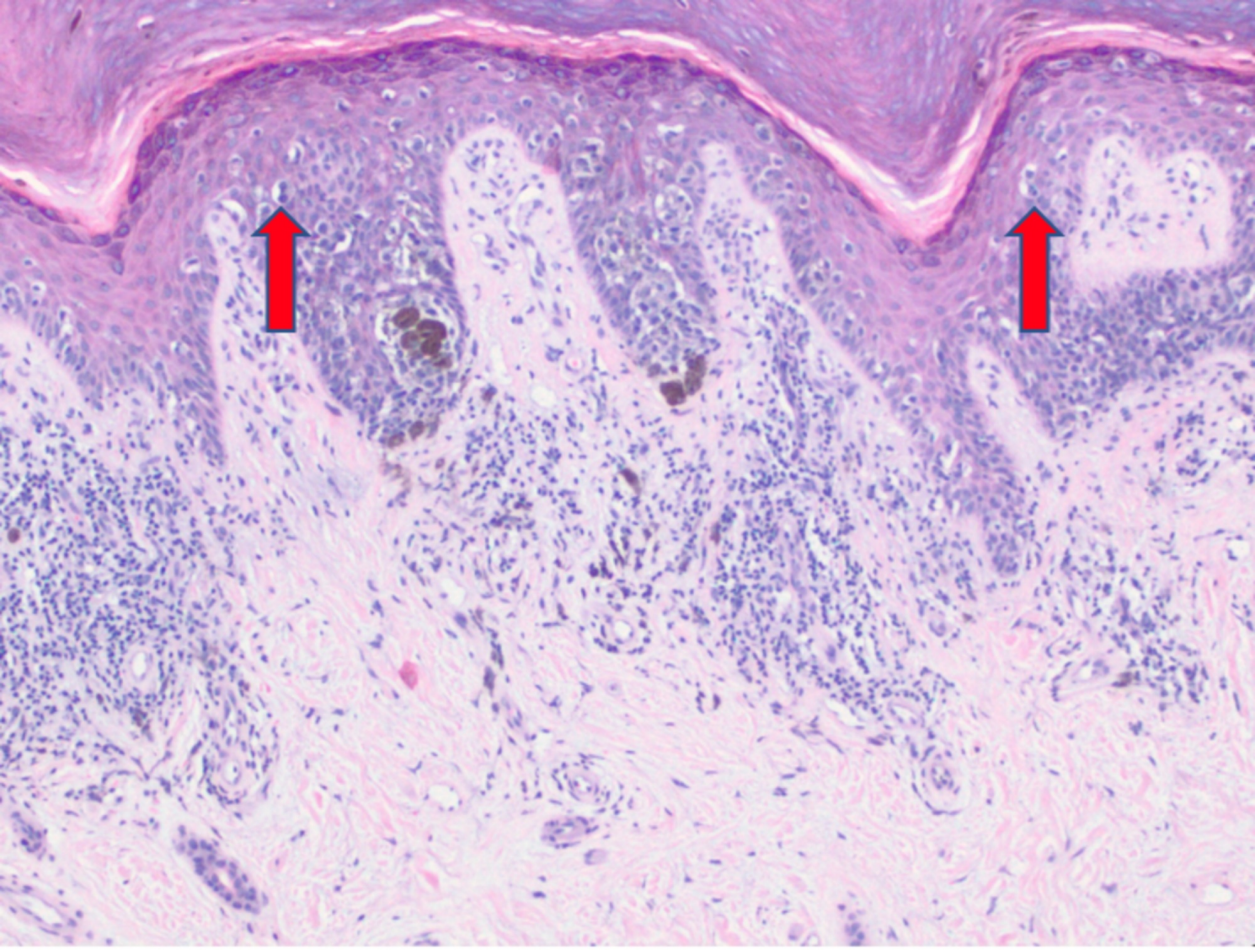
Extensive proliferation of malignant melanocytes in a pagetoid array (10x).

**Figure 5:**
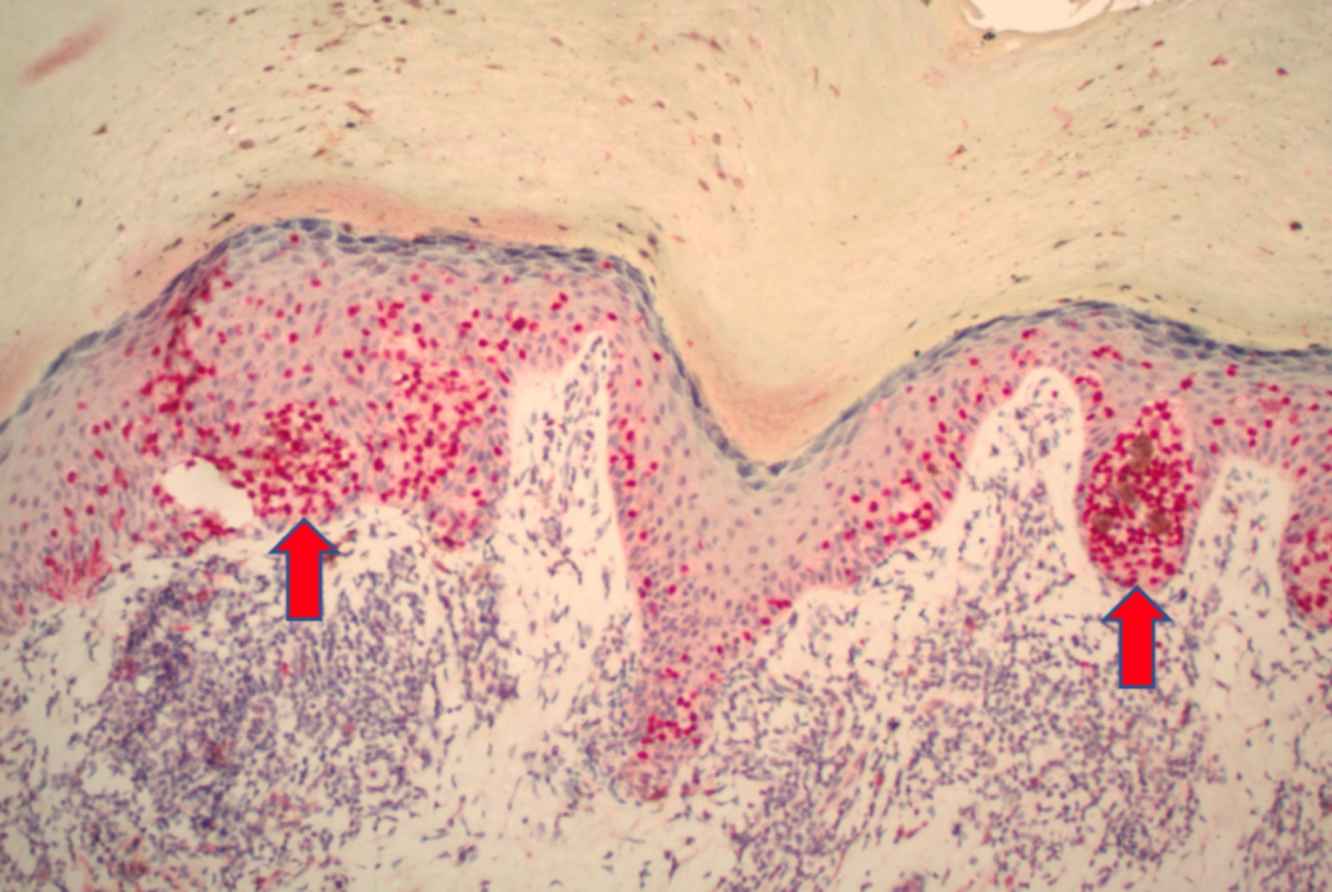
SOX10 immunohistochemical stain demonstrating extensive proliferation of melanocytes within the epidermis (10x).

Multiple shave biopsies were performed to remove all the clinical pigment. Pathology revealed extensive proliferation of malignant melanocytes in a lentiginous, nested, and pagetoid array (Figures 2-4). These findings are consistent with malignant melanoma in situ, acral lentiginous type with confirmation of diagnosis with SOX10 immunohistochemical stain (Figure 5).

The patient underwent wide local excision, under general anesthesia, to the level of underlying fascia with 1 cm margins. An acellular allograft dermal matrix was utilized to close the defect created by lesion removal. Surgical margins were negative for residual ALM. The patient recovered from surgery without complications and no further treatment was required. The patient was instructed to follow with dermatology for full body skin exams every three months. 

## Discussion

Acral lentiginous melanoma represents approximately 5% of melanomas diagnosed each year. It is found on the palms, soles, and in association with the nail unit [2]. While the incidence of ALM is similar in all racial and ethnic groups, ALM represents a disproportionate percentage of melanomas in darker-skinned individuals. This may be due to the unique pathophysiology of ALM, as these lesions often develop on sun-protected areas. This is in contrast to other forms of melanoma, in which ultraviolet-B (UVB) exposure is a well-known risk factor for development [1].

Clinically, ALM of the palmar or plantar surface presents as a pigmented macule or patch, with varying shades of brown to black, as was seen in our patient [3]. Lesions are typically larger than 7.0 mm in diameter and are irregularly shaped or asymmetrical, with notching at the periphery [3]; ALM may lack macroscopic pigmentation [4]. The distinction between benign acral nevus and ALM may be facilitated with the utilization of dermoscopy. Perhaps the most crucial difference between ALM and benign nevi of acral surfaces is the parallel band-like pigmentation on the ridges of the surface skin markings seen in early acral melanoma [3]. In contrast, pigmentation is noted in the skin furrows of benign acral nevi [3]. ALM originating in the nail matrix may present as longitudinal melanonychia, hyperpigmentation of proximal or lateral nail fold or hyponychium, or in an amelanotic fashion that often mimics a pyogenic granuloma [1].

Histologically, ALM often displays a lentiginous growth pattern with nests of melanocytes arranged in units along the basal layer of the epidermis at the dermal-epidermal junction. Pagetoid spread may be absent in the early stage and is known to occur later [5]. A vertical growth phase may also exist in some cases of ALM and histopathologically one would expect to see a central plaque-like thickening of malignant melanocytes extending into the papillary dermis, with concurrent dysplasia of spindle cells, epidermal hyperplasia, and elongation of the rete ridges [6]. The vertical growth phase may be visualized clinically by a nodular component to the lesion.

In contrast to other forms of melanoma in which UVB exposure is a known risk factor for the development of melanoma, the connection between UVB as a pathogenic driver of ALM is not as clear [7]. However, expression of the c-KIT gene is known to play a role in the development of ALM [8]. Specifically, an activating mutation in the c-KIT gene leads to excessive differentiation and proliferation of melanocytes in ALM with up to 84% of melanocytes expressing the KIT protein in one study [8].

Surgical excision is the treatment of choice for most patients with ALM, as it was for our patient, and is generally curative. For patients with advanced-stage disease or metastases that are not surgically resectable, treatment options include BRAF inhibitors alone or in combination with MEK inhibitors such as vemurafenib and cobemetinib respectively, both of which have shown promising results [9]. KIT inhibitors, like imatinib, may be particularly suited to treat ALM due to the activating mutation in c-KIT [1]. Other treatment options include immune checkpoint inhibitors and cell death regulators targeting anti-CTLA4 and anti-PD1 antibodies [9]. One study recently found the histone variant H2A.Z.2 and a related protein, BRD2, to be integral to the pathogenesis of malignant melanoma and more specifically, metastatic melanoma through transcriptional regulation of E2F genes on melanocytes [10]. Future treatment options may include biologics that target these two proteins in an attempt to minimize the proliferation of malignant melanocytes to stop the growth of the primary cancer or limit metastatic spread.

While available treatment options for cutaneous melanomas continue to improve, plantar melanomas, the most common site of involvement in non-Caucasian patients, are often associated with a poor prognosis [7]. While the explanation for this is likely multi-factorial, one important factor is that non-Caucasian ethnic groups tend to present with more advanced tumors than Caucasians patients [7]. Reasons for this may include less access to preventative screenings and a misconception that darker races do not develop skin cancer [7]. Since 1974, visits to a dermatologist in the United States have almost doubled, but only 8% of these patients were non-Caucasian patients [11]. This may be, in part, due to normal variations like longitudinal melanonychia and hyperpigmented macules of palms and soles in patients with skin of color. Patients, and many healthcare providers, may not be equipped to discern the difference between these benign entities and ALM. For this reason, patients may not present for dermatological evaluation at earlier stages of the disease due to the assumption that their lesion is benign. Current public health education programs for skin cancer are directed towards Caucasian patients, specifically high-risk individuals with fair skin and light eyes [7]. Physician training, patient education, and public awareness campaigns directed toward all ethnic groups may decrease time to diagnosis for ALM and, therefore, lead to a more favorable prognosis.

## Conclusions

Acral lentiginous melanoma is a rare subtype of malignant melanoma, generally found on the palms, soles, and subungual spaces. ALM is the most common subtype of melanoma diagnosed in non-Caucasian patients. Careful clinical, dermoscopic, and histopathological evaluation is required to accurately diagnose these melanomas at an early stage. Our case highlights an ALM in an FST 5 female that had been present for 10 years before initial evaluation. Fortunately, at the time of presentation, this lesion was “in situ” and was able to be treated with wide local excision. However, delayed evaluation and diagnosis do not always yield such favorable results. We propose physician training and public health efforts to increase awareness of this rare type of melanoma to increase the number of non-Caucasian patients who are evaluated by dermatologists each year. Therefore, this may decrease time to diagnosis for ALM, resulting in a better prognosis.
